# Supplementary material for: Improving model fairness in image-based computer-aided diagnosis
Source: Nat Commun. 2023 Oct 6;14:6261. doi: 10.1038/s41467-023-41974-4 (PMC10558498; doi:10.1038/s41467-023-41974-4)
Supplement: Supplementary file 1 — Supplementary Information [file 41467_2023_41974_MOESM1_ESM.pdf]

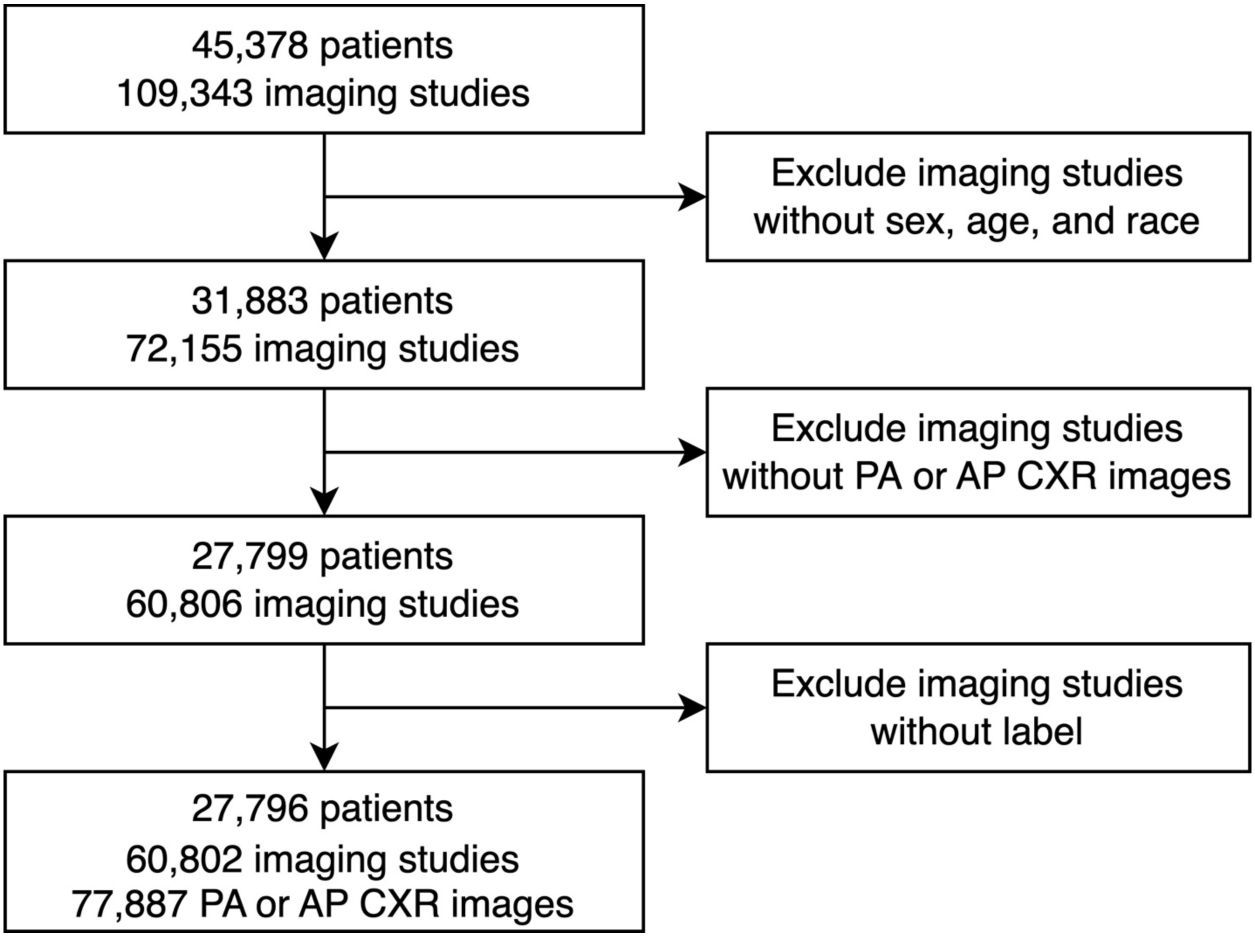

Supplementary Figure 1. Creation of MIDRC dataset.

Supplementary Table 1. The AUCs and Pairwise Fairness Difference (PFD) of baseline, baseline with oversampling (Balance baseline), and proposed model for the age subgroup in the MIDRC dataset.

Age	Baseline	Balance Baseline	Proposed model
Overall	0.8216 ± 0.0165	0.8106 ± 0.0152	0.8052 ± 0.0177
< 75 years	0.8324 ± 0.0171	0.8217 ± 0.0155	0.8117 ± 0.0191
>= 75 years	0.7552 ± 0.0212	0.7412 ± 0.0186	0.7657 ± 0.0120
PFD	0.0773 ± 0.0193	0.0805 ± 0.0146	0.0462 ± 0.0143

Supplementary Table 2. The AUCs and Pairwise Fairness Difference (PFD) of baseline, baseline with oversampling (Balance baseline), and proposed model for the sex subgroup in the MIDRC dataset.

Sex	Baseline	Balance Baseline	Proposed model
Overall	0.8216 ± 0.0165	0.8218 ± 0.0171	0.8137 ± 0.0167
Male	0.8195 ± 0.0207	0.8201 ± 0.0201	0.8117 ± 0.0164
Female	0.8244 ± 0.0140	0.8241 ± 0.0143	0.8166 ± 0.0168
PFD	0.0106 ± 0.0108	0.0097 ± 0.0100	0.0049 ± 0.0019

Supplementary Table 3. The AUCs and Pairwise Fairness Difference (PFD) of baseline, baseline with oversampling (Balance baseline), and proposed model for the race subgroup in the MIDRC dataset.

Race	Baseline	Balance Baseline	Proposed model
Overall	0.8216 ± 0.0165	0.8110 ± 0.0163	0.8016 ± 0.0150
White	0.7722 ± 0.0216	0.7646 ± 0.0231	0.7731 ± 0.0157
Black	0.8704 ± 0.0153	0.8554 ± 0.0152	0.8293 ± 0.0219
Other races	0.7319 ± 0.0485	0.7379 ± 0.0464	0.7497 ± 0.0375
PFD	0.1386 ± 0.0504	0.1174 ± 0.0347	0.0835 ± 0.0456

Supplementary Table 4. The AUCs and Pairwise Fairness Difference (PFD) of baseline, baseline with oversampling (Balance baseline), and proposed model for the age subgroup in the MIMIC-CXR dataset.

Age	Baseline	Balance Baseline	Proposed model
Overall	0.7818 ± 0.0076	0.7839 ± 0.0048	0.7818 ± 0.0041
< 60 years	0.8370 ± 0.0119	0.8428 ± 0.0097	0.8172 ± 0.0107
>= 60 years	0.7524 ± 0.0083	0.7525 ± 0.0045	0.7629 ± 0.0038
PFD	0.0845 ± 0.0126	0.0903 ± 0.0101	0.0543 ± 0.0119

Supplementary Table 5. The AUCs and Pairwise Fairness Difference (PFD) of baseline, baseline with oversampling (Balance baseline), and proposed model for the sex subgroup in the MIMIC-CXR dataset.

Sex	Baseline	Balance Baseline	Proposed model
Overall	0.7818 ± 0.0076	0.7793 ± 0.0034	0.7761 ± 0.0070
Male	0.7594 ± 0.0098	0.7493 ± 0.0101	0.7616 ± 0.0069
Female	0.8041 ± 0.0142	0.8091 ± 0.0140	0.7905 ± 0.0135
PFD	0.0447 ± 0.0189	0.0598 ± 0.0236	0.0289 ± 0.0163

Supplementary Table 6. The AUCs and Pairwise Fairness Difference (PFD) of baseline, baseline with oversampling (Balance baseline), and proposed model for the race subgroup in the MIMIC-CXR dataset.

Sex	Baseline	Balance Baseline	Proposed model
Overall	0.7818 ± 0.0076	0.7717 ± 0.7689	0.7724 ± 0.0056
Other races	0.8076 ± 0.0039	0.7899 ± 0.075	0.8063 ± 0.0072
Black	0.7770 ± 0.0089	0.7649 ± 0.0085	0.7659 ± 0.0055
PFD	0.0307 ± 0.0089	0.0250 ± 0.0080	0.0404 ± 0.0033

Supplementary Table 7. The AUCs and Pairwise Fairness Difference (PFD) of baseline, baseline with oversampling (Balance baseline), and proposed model for the age subgroup in the OHTS dataset.

Age	Baseline	Balance Baseline	Proposed model
Overall	0.8892 ± 0.0137	0.9184 ± 0.0100	0.9019 ± 0.0171
< 60 years	0.8389 ± 0.0335	0.8922 ± 0.0328	0.8793 ± 0.0186
>= 60 years	0.8994 ± 0.0133	0.9229 ± 0.0092	0.9064 ± 0.0186
PFD	0.0605 ± 0.0390	0.0383 ± 0.0239	0.0279 ± 0.0222

Supplementary Table 8. The AUCs and Pairwise Fairness Difference (PFD) of baseline, baseline with oversampling (Balance baseline), and proposed model for the sex subgroup in the OHTS dataset.

Sex	Baseline	Balance Baseline	Proposed model
Overall	0.8892 ± 0.0137	0.9063 ± 0.0253	0.8956 ± 0.0197
Male	0.9032 ± 0.0063	0.9175 ± 0.0154	0.9028 ± 0.0122
Female	0.8730 ± 0.0336	0.8914 ± 0.0450	0.8842 ± 0.0336
PFD	0.0302 ± 0.0300	0.0302 ± 0.0311	0.0194 ± 0.0221

Supplementary Table 9. The AUCs and Pairwise Fairness Difference (PFD) of baseline, baseline with oversampling (Balance baseline), and proposed model for the race subgroup in the OHTS dataset.

Race	Baseline	Balance Baseline	Proposed model
Overall	0.8892 ± 0.0137	0.8986 ± 0.0250	0.8922 ± 0.0111
Other races	0.8845 ± 0.0198	0.8946 ± 0.0493	0.8906 ± 0.0130
Black	0.8941 ± 0.0233	0.9017 ± 0.0209	0.8980 ± 0.0144
PFD	0.0228 ± 0.0257	0.0324 ± 0.0185	0.0128 ± 0.0072

Supplementary Table 10. The AUCs and Pairwise Fairness Difference (PFD) of baseline, baseline with oversampling (Balance baseline), and proposed model for the age subgroup in the AREDS dataset.

Race	Baseline	Balance Baseline	Proposed model
Overall	0.9808 ± 0.0029	09812 ± 0.0030	0.9810 ± 0.0019
< 65	0.9575 ± 0.0230	0.9546 ± 0.0200	0.9634 ± 0.0230
65-75	0.9745 ± 0.0073	0.9742 ± 0.0058	0.9766 ± 0.0048
>= 75	0.9851 ± 0.0029	0.9860 ± 0.0034	0.9843 ± 0.0014
PFD	0.0302 ± 0.0207	0.0323 ± 0.0212	0.0226 ± 0.0153

Supplementary Table 11. The AUCs and Pairwise Fairness Difference (PFD) of baseline, baseline with oversampling (Balance baseline), and proposed model for the sex subgroup in the AREDS dataset.

Sex	Baseline	Balance Baseline	Proposed model
Overall	0.9808 ± 0.0029	0.9823 ± 0.0025	0.9814 ± 0.0029
Male	0.9808 ± 0.0029	0.9832 ± 0.0043	0.9814 ± 0.0029
Female	0.9818 ± 0.0054	0.9816 ± 0.0023	0.9828 ± 0.0051
PFD	0.0034 ± 0.0033	0.0032 ± 0.0027	0.0036 ± 0.0030

Supplementary Table 12. The AUCs and Pairwise Fairness Difference (PFD) of baseline, baseline with oversampling (Balance baseline), and proposed model for the CFH (rs1061170) subgroup in the AREDS dataset.

Race	Baseline	Balance Baseline	Proposed model
Overall	0.9795 ± 0.0040	0.9781 ± 0.0063	0.9810 ± 0.0051
TT	0.9720 ± 0.0145	0.9655 ± 0.0206	0.9734 ± 0.0141
CT	0.9781 ± 0.0029	0.9765 ± 0.0043	0.9792 ± 0.0039
CC	0.9856 ± 0.0047	0.9851 ± 0.0054	0.9863 ± 0.0038
PFD	0.0198 ± 0.0115	0.0210 ± 0.0178	0.0141 ± 0.0093

Supplementary Table 13. The AUCs and Pairwise Fairness Difference (PFD) of baseline, baseline with oversampling (Balance baseline), and proposed model for the ARMS2 (rs10490924) subgroup in the AREDS dataset.

Race	Baseline	Balance Baseline	Proposed model
Overall	0.9795 ± 0.0040	0.9808 ± 0.0051	0.9810 ± 0.0047
GG	0.9757 ± 0.0048	0.9760 ± 0.0075	0.9787 ± 0.0046
GT	0.9792 ± 0.0029	0.9805 ± 0.0037	0.9804 ± 0.0051
TT	0.9848 ± 0.0117	0.9868 ± 0.0089	0.9855 ± 0.0071
PFD	0.0150 ± 0.0026	0.0130 ± 0.0068	0.0076 ± 0.0054

Supplementary Table 14. The AUCs and Pairwise Fairness Difference (PFD) of baseline, baseline with oversampling (Balance baseline), and proposed model for the age-race intersectional group in the MIDRC dataset.

Age	Baseline	Balance Baseline	Proposed model
Overall	0.8216 ± 0.0165	0.8081 ± 0.0178	0.8007 ± 0.0120
White older	0.7035 ± 0.0276	0.6873 ± 0.0196	0.7326 ± 0.0178
White younger	0.8001 ± 0.0417	0.7770 ± 0.0272	0.7826 ± 0.0174
Black older	0.8334 ± 0.0313	0.8178 ± 0.0126	0.8130 ± 0.0205
Black younger	0.8748 ± 0.0135	0.8562 ± 0.0158	0.8302 ± 0.0084
Other older	0.6893 ± 0.0759	0.7017 ± 0.0930	0.7532 ± 0.0583
Other younger	0.7416 ± 0.0576	0.7466 ±0.0458	0.7490 ± 0.0536
PFD	0.2104 ± 0.0416	0.1967 ± 0.0391	0.1101 ± 0.0297

Supplementary Table 15. The AUCs and Pairwise Fairness Difference (PFD) of baseline, baseline with oversampling (Balance baseline), and proposed model for the age-sex intersectional group in the MIMIC-CXR dataset.

Age-sex	Baseline	Balance Baseline	Proposed model
Overall	0.7818 ± 0.0076	0.7798 ± 0.0058	0.7774 ± 0.0068
Younger male	0.8135 ± 0.0074	0.8089 ± 0.0185	0.7991 ± 0.0189
Younger female	0.7364 ± 0.0131	0.7281 ± 0.0131	0.7361 ± 0.0097
Older male	0.8564 ± 0.0203	0.8626 ± 0.0092	0.8408 ± 0.0183
Older female	0.7710 ± 0.0114	0.7707 ± 0.0050	0.7728 ± 0.0179
PFD	0.1180 ± 0.0279	0.1345 ± 0.0206	0.1020 ±0.0263

Supplementary Table 16. The AUCs and Pairwise Fairness Difference (PFD) of baseline, baseline with oversampling (Balance baseline), and proposed model for the age-sex intersectional group in the OHTS dataset.

Age-sex	Baseline	Balance Baseline	Proposed model
Overall	0.8892 ± 0.0137	0.9073 ± 0.0212	0.8919 ± 0.0156
Younger male	0.8746 ± 0.0586	0.9135 ± 0.0412	0.8709 ± 0.0195
Younger female	0.7896 ± 0.0603	0.8249 ± 0.0512	0.8367 ± 0.0471
Older male	0.9078 ± 0.0154	0.9306 ± 0.0187	0.9072 ± 0.0134
Older female	0.8844 ± 0.0249	0.8959 ± 0.0418	0.8897 ± 0.0357
PFD	0.1298 ± 0.0505	0.1165 ± 0.0450	0.0842 ± 0.0387

Supplementary Table 17. The AUCs and Pairwise Fairness Difference (PFD) of baseline, baseline with oversampling (Balance baseline), and proposed model for the age-CFH intersectional group in the AREDS dataset.

Age-cfh	Baseline	Balance Baseline	Proposed model
Overall	0.9808 ± 0.0029	0.9784 ± 0.0043	0.9802 ± 0.0046
< 60 years old and CFH = TT	0.8647 ± 0.1345	0.8808 ± 0.1058	0.8896 ± 0.1252
< 60 years old and CFH = CT	0.9271 ± 0.0677	0.9460 ± 0.0561	0.9362 ± 0.0447
< 60 years old and CFH = CC	0.9823 ± 0.0079	0.9770 ± 0.0233	0.9761 ± 0.0205
60-75 years old and CFH = TT	0.9577 ± 0.0203	0.9614 ± 0.0131	0.9630 ± 0.0047
60-75 years old and CFH = CT	0.9735 ± 0.0088	0.9608 ± 0.0215	0.9761 ± 0.0099
60-75 years old and CFH = CC	0.9837 ± 0.0083	0.9778 ± 0.0078	0.9844 ± 0.0080
>=75 years old and CFH = TT	0.9838 ± 0.0093	0.9820 ± 0.0074	0.9849 ± 0.0081
>=75 years old and CFH = CT	0.9828 ± 0.0064	0.9799 ± 0.0061	0.9817 ± 0.0040
>=75 years old and CFH = CC	0.9848 ± 0.0056	0.9882 ± 0.0030	0.9874 ± 0.0042
PFD	0.1580 ± 0.1167	0.1257 ± 0.0907	0.1131 ± 0.1132

Supplementary Table 18. The AUCs and Pairwise Fairness Difference (PFD) of baselineand proposed model for the age subgroup in the MIDRC dataset based on ResNet-152.

Age	Baseline	Proposed model
Overall	0.8168 ± 0.0233	0.8116 ± 0.0239
< 75 years	0.8231 ± 0.0215	0.8154 ± 0.0250
>= 75 years	0.7569 ± 0.0151	0.7896 ± 0.0227
PFD	0.0660 ± 0.0191	0.0258 ± 0.0176

Supplementary Table 19. The AUCs and Pairwise Fairness Difference (PFD) of baseline and proposed model for the sex subgroup in the MIDRC dataset based on ResNet-152.

Sex	Baseline	Proposed model
Overall	0.8169 ± 0.0233	0.8155 ± 0.0219
Male	0.8144 ± 0.0311	0.8106 ± 0.0265
Female	0.8203 ± 0.0157	0.8221 ± 0.0159
PFD	0.0176 ± 0.0113	0.01324 ± 0.0088

Supplementary Table 20. The AUCs and Pairwise Fairness Difference (PFD) of baseline and proposed model for the race subgroup in the MIDRC dataset based on ResNet-152.

Race	Baseline	Proposed model
Overall	0.8169 ± 0.0233	0.8072 ± 0.0138
White	0.7793 ± 0.0269	0.7839 ± 0.0156
Black	0.8575 ± 0.0227	0.8322 ± 0.0183
Other races	0.7296 ± 0.0466	0.7513 ± 0.0366
PFD	0.1279 ± 0.0458	0.0809 ± 0.0451

Supplementary Table 21. The AUCs and Pairwise Fairness Difference (PFD) of baseline and proposed model for the age subgroup in the OHTS dataset based on ResNet-152.

Age	Baseline	Proposed model
Overall	0.8806 ± 0.0146	0.9141 ± 0.0195
< 60 years	0.8521 ± 0.0303	0.8829 ± 0.0243
>= 60 years	0.8878 ± 0.0185	0.9206 ± 0.0173
PFD	0.0434 ± 0.0286	0.0377 ± 0.0159

Supplementary Table 22. The AUCs and Pairwise Fairness Difference (PFD) of baseline and proposed model for the sex subgroup in the OHTS dataset based on ResNet-152.

Sex	Baseline	Proposed model
Overall	0.8806 ± 0.0146	0.9044 ± 0.0154
Male	0.8956 ± 0.0168	0.9141 ± 0.0108
Female	0.8596 ± 0.0311	0.8880 ± 0.0298
PFD	0.0359 ± 0.0395	0.0290 ± 0.0247

Supplementary Table 23. The AUCs and Pairwise Fairness Difference (PFD) of baseline and proposed model for the race subgroup in the OHTS dataset based on ResNet-152.

Sex	Baseline	Proposed model
Overall	0.8806 ± 0.0146	0.9059 ± 0.0136
Other races	0.8700 ± 0.0316	0.8960 ± 0.0192
Black	0.8804 ± 0.0252	0.9297 ± 0.0209
PFD	0.0409 ± 0.0027	0.0356 ± 0.0270

Supplementary Table 24. The AUCs and Pairwise Fairness Difference (PFD) of baseline and proposed model for the age-race intersectional group in the MIDRC dataset based on ResNet-152.

Age	Baseline	Proposed model
Overall	0.8169 ± 0.0233	0.7990 ± 0.0144
White older	0.7206 ± 0.0496	0.7700 ± 0.0156
White younger	0.7930 ± 0.0318	0.7798 ± 0.0222
Black older	0.8205 ± 0.0338	0.8314 ± 0.0147
Black younger	0.8623 ± 0.0215	0.8201 ± 0.0137
Other older	0.6801 ± 0.0737	0.8201 ± 0.0466
Other younger	0.7412 ± 0.0559	0.7458 ± 0.0550
PFD	0.1940 ± 0.0544	0.1089 ± 0.0478

Supplementary Table 25. The AUCs and Pairwise Fairness Difference (PFD) of baseline and proposed model for the age-sex intersectional group in the OHTS dataset based on ResNet-152.

Age-sex	Baseline	Proposed model
Overall	0.8806 ± 0.0146	0.9052 ± 0.0175
Younger male	0.8800 ± 0.0335	0.8747 ± 0.0458
Younger female	0.8084 ± 0.1114	0.8523 ± 0.0376
Older male	0.9016 ± 0.0284	0.9171 ± 0.0192
Older female	0.8699 ± 0.0181	0.9075 ± 0.0256
PFD	0.0359 ± 0.0395	0.0290 ± 0.0245

Supplementary Table 26. The characteristics of AREDS on two genes.

Disease	Dataset	Subgroup	Attribute	Positive		Total
					%	
AMD	AREDS		No. of images	5,433	11.75	46,244
		CHF	TT	906	6.17	14,679
			CT	2,401	11.40	21,059
			CC	2,126	20.24	10,506
		ARMS2	GG	1,558	6.44	24,206
			GT	2,514	14.45	17,399
			TT	1,361	29.34	4,639
