# Supplementary material for: Improving model fairness in image-based computer-aided diagnosis
Source: Nat Commun. 2023 Oct 6;14:6261. doi: 10.1038/s41467-023-41974-4 (PMC10558498; doi:10.1038/s41467-023-41974-4)
Supplement: Supplementary file 3 — Reporting Summary [file 41467_2023_41974_MOESM3_ESM.pdf]

nature portfolio

Corresponding author(s): Yifan Peng

Last updated by author(s): Aug 7, 2023

Reporting Summary

Nature Portfolio wishes to improve the reproducibility of the work that we publish. This form provides structure for consistency and transparency in reporting. For further information on Nature Portfolio policies, see our Editorial Policies and the Editorial Policy Checklist.

Statistics

For all statistical analyses, confirm that the following items are present in the figure legend, table legend, main text, or Methods section.

n/a	Confirmed	
☐	☒	The exact sample size (*n*) for each experimental group/condition, given as a discrete number and unit of measurement
☐	☒	A statement on whether measurements were taken from distinct samples or whether the same sample was measured repeatedly
☒	☐	The statistical test(s) used AND whether they are one- or two-sided<br>*Only common tests should be described solely by name; describe more complex techniques in the Methods section.*
☐	☒	A description of all covariates tested
☒	☐	A description of any assumptions or corrections, such as tests of normality and adjustment for multiple comparisons
☐	☒	A full description of the statistical parameters including central tendency (e.g. means) or other basic estimates (e.g. regression coefficient) AND variation (e.g. standard deviation) or associated estimates of uncertainty (e.g. confidence intervals)
☒	☐	For null hypothesis testing, the test statistic (e.g. *F*, *t*, *r*) with confidence intervals, effect sizes, degrees of freedom and *P* value noted<br>*Give P values as exact values whenever suitable.*
☒	☐	For Bayesian analysis, information on the choice of priors and Markov chain Monte Carlo settings
☒	☐	For hierarchical and complex designs, identification of the appropriate level for tests and full reporting of outcomes
☒	☐	Estimates of effect sizes (e.g. Cohen's *d*, Pearson's *r*), indicating how they were calculated

Our web collection on statistics for biologists contains articles on many of the points above.

Software and code

Policy information about availability of computer code

Data collection	We use Gen 3 version 2022.10 to download MIDRC and don't need to use any specific software to download the other three datasets.
Data analysis	python >=3.6, pytorch = 1.11.0, torchvision = 0.12.0, scikit-learn = 0.23.2, pandas = 1.4.1, tqdm = 4.48.2, opencv = 4.5.0, skimage = 0.17.2, json = 0.9.6, pickle = 2.2.1R version 2022.12.0+353. Codes are available at doi.org/10.5281/zenodo.8226443.

For manuscripts utilizing custom algorithms or software that are central to the research but not yet described in published literature, software must be made available to editors and reviewers. We strongly encourage code deposition in a community repository (e.g. GitHub). See the Nature Portfolio guidelines for submitting code & software for further information.

Data

Policy information about availability of data

All manuscripts must include a data availability statement. This statement should provide the following information, where applicable:

- Accession codes, unique identifiers, or web links for publicly available datasets
- A description of any restrictions on data availability
- For clinical datasets or third party data, please ensure that the statement adheres to our policy

The first dataset is publicly available at Medical Imaging and Data Resource Center (https://data.midrc.org/explorer). The AREDS dataset is publicly available on NCBI dbGAP (https://www.ncbi.nlm.nih.gov/projects/gap/cgibin/study.cgi?study_id=phs000001.v3.p1). The OHTS dataset is available upon request due to patient protection (https://ohts.wustl.edu/). The MIMIC-CXR dataset is publicly available on PhysioNet (https://www.physionet.org/content/mimic-cxr-jpg/).

Research involving human participants, their data, or biological material

Policy information about studies with human participants or human data. See also policy information about sex, gender (identity/presentation), and sexual orientation and race, ethnicity and racism.

Reporting on sex and gender	*Use the terms sex (biological attribute) and gender (shaped by social and cultural circumstances) carefully in order to avoid confusing both terms. Indicate if findings apply to only one sex or gender; describe whether sex and gender were considered in study design; whether sex and/or gender was determined based on self-reporting or assigned and methods used. Provide in the source data disaggregated sex and gender data, where this information has been collected, and if consent has been obtained for sharing of individual-level data; provide overall numbers in this Reporting Summary. Please state if this information has not been collected. Report sex- and gender-based analyses where performed, justify reasons for lack of sex- and gender-based analysis.*
Reporting on race, ethnicity, or other socially relevant groupings	*Please specify the socially constructed or socially relevant categorization variable(s) used in your manuscript and explain why they were used. Please note that such variables should not be used as proxies for other socially constructed/relevant variables (for example, race or ethnicity should not be used as a proxy for socioeconomic status). Provide clear definitions of the relevant terms used, how they were provided (by the participants/respondents, the researchers, or third parties), and the method(s) used to classify people into the different categories (e.g. self-report, census or administrative data, social media data, etc.) Please provide details about how you controlled for confounding variables in your analyses.*
Population characteristics	*Describe the covariate-relevant population characteristics of the human research participants (e.g. age, genotypic information, past and current diagnosis and treatment categories). If you filled out the behavioural & social sciences study design questions and have nothing to add here, write "See above."*
Recruitment	*Describe how participants were recruited. Outline any potential self-selection bias or other biases that may be present and how these are likely to impact results.*
Ethics oversight	*Identify the organization(s) that approved the study protocol.*

Note that full information on the approval of the study protocol must also be provided in the manuscript.

Field-specific reporting

Please select the one below that is the best fit for your research. If you are not sure, read the appropriate sections before making your selection.

☐ Life sciences ☒ Behavioural & social sciences ☐ Ecological, evolutionary & environmental sciences

For a reference copy of the document with all sections, see nature.com/documents/nr-reporting-summary-flat.pdf

Behavioural & social sciences study design

All studies must disclose on these points even when the disclosure is negative.

Study description	In this study, we introduce an approach to reduce bias towards groups in deep learning models for image-based computer-aided diagnosis while preserving the overall performance. The data are quantitative.
Research sample	Four public datasets were used in this study, the patient number of these four datasets are 27,779, 227,827, 1,636, and 4,566 for MIDRC, MIMIC-CXR, OTHS, and AREDS, respectively. MIDRC data is a chest X-ray imaging repository that was specifically created for COVID-19 diagnosis. The repository is part of the Medical Imaging and Data Resource Center (MIDRC), which is a collaborative initiative involving multiple institutions and is funded by the National Institute of Biomedical Imaging and Bioengineering (NIBIB) under contracts 75N92020C00008 and 75N92020C00021 and hosted at the University of Chicago. MIDRC is coled by the American College of Radiology® (ACR®), the Radiological Society of North America (RSNA), and the American Association of Physicists in Medicine (AAPM). The Age-Related Eye Disease Studies (AREDS) cohort was a 12-year multi-center prospective study sponsored by the National Eye Institute (National Institutes of Health) that investigated the clinical course, prognosis, and risk factors of age-related macular degeneration (AMD). Ocular Hypertension Treatment Study (OHTS) is a large longitudinal clinical trial with 1,636 participants and 37,399 images collected from 22 centers in the United States investigating conversion to primary open-angle glaucoma (POAG) in eyes with elevated intraocular pressure. MIMIC-CXR is a large public dataset of 377,110 chest x-rays associated with 227,827 patients presenting to the Beth Israel Deaconess Medical Center Emergency Department between 2011–2016. The samples of the studies are representative. Moreover, all four datasets are publicly available, enabling other researchers to utilize the data. Additionally, each of these datasets represents one of the largest collections of data for their respective diseases and includes valuable demographic information. Last but not least, these datasets are sourced from diverse regions, contributing to their broader applicability and relevance in medical research.
Sampling strategy	We used public dataset in this study, and we use all the available data in the datasets. For the MIDRC, ARDES, and OHTS datasets, the entire data set was randomly split at the patient level. One group (20% of the total subjects) was used as the hold-out test set and the remaining as the training set. For the MIMIC-CXR dataset, the official release training, validation, and testing datasets were used.
Data collection	This study uses existing datasets. New new data was collected. We downloaded MIDRC dataset through this website (https://data.midrc.org/explorer). We downloaded AREDS dataset from NCBI dbGAP (https://www.ncbi.nlm.nih.gov/projects/gap/cgibin/

	study.cgi?study_id=phs000001.v3.p1). We downloaded OHTS dataset from https://ohts.wustl.edu/. We downloaded MIMIC-CXR dataset from PhysioNet (https://www.physionet.org/content/mimic-cxr-jpg/). We were not blinded to experimental condition.
Timing	We downloaded the MIDRC dataset on April 2023, OHTS dataset on February 2021, AREDS dataset on October 2022, and MIMIC-CXR on July 2022.
Data exclusions	We did not exclude data after data collection.
Non-participation	We used public dataset in this study, so there are no participant involved in this study.
Randomization	We used all the available data in the datasets. For the MIDRC, ARDES, and OHTS datasets, the entire data set was randomly split at the patient level. One group (20% of the total subjects) was used as the hold-out test set and the remaining as the training set. For the MIMIC-CXR dataset, the official release training, validation, and testing datasets were used.

Reporting for specific materials, systems and methods

We require information from authors about some types of materials, experimental systems and methods used in many studies. Here, indicate whether each material, system or method listed is relevant to your study. If you are not sure if a list item applies to your research, read the appropriate section before selecting a response.

Materials & experimental systems

n/a	Involved in the study	
☒	☐	Antibodies
☒	☐	Eukaryotic cell lines
☒	☐	Palaeontology and archaeology
☒	☐	Animals and other organisms
☒	☐	Clinical data
☒	☐	Dual use research of concern
☒	☐	Plants

Methods

n/a	Involved in the study	
☒	☐	ChIP-seq
☒	☐	Flow cytometry
☒	☐	MRI-based neuroimaging
